# Prevalence of thyroid disorders among children with Down syndrome seen in the out-patient clinics of the Philippine general hospital

**DOI:** 10.1186/1687-9856-2015-S1-P101

**Published:** 2015-04-28

**Authors:** Lalaine Audrey Matitu-Untalan, Sylvia C Estrada

**Affiliations:** 1 Philippine General Hospital, Manila, Philippines

## Background

Thyroid disorders are noted to occur in 28–40% of children with Down syndrome [1]. Hypothyroidism has a subtle presentation and can be particularly challenging to detect in patients with intellectual disabilities and communication and language impairments [2]. Regular screening and early diagnosis of thyroid disorders, particularly hypothyroidism, is essential for early intervention.

## Objective

To determine the prevalence of thyroid disorders among children with Down Syndrome (DS) seen in the outpatient clinics of the Philippine General Hospital from January 2007 to December 2011.

## Secondary objectives

To classify the thyroid disorders present; to describe the clinical profiles and genotype of the children with Down syndrome.

## Methodology

A Retrospective chart review of all patients with Down syndrome seen in the Out-patient Clinics of the Philippine General Hospital from January 2007 to December 2011.

## Results

Eighty-nine patients were included, 60% males and 40% females .Fifty-six percent had thyroid disorders. Of these, eighty percent had subclinical hypothyroidism, 12% had overt hypothyroidism, while 8% had hyperthyroidism. The mean age for the diagnosis of thyroid disorder was 3.33 (±0.52) years old. The mean age on initial consult was 2.38 (±3.14) years. The mean age of mothers at childbirth was 34.23 (±6.77) years. The most common co-morbid illness was congenital heart disease (46%, 41/89). The most common chromosomal abnormality was full trisomy 21 (95.51%, 85/89).

## Conclusion

Fifty-six percent of children with Down syndrome in this study have thyroid disorders, with subclinical hypothyroidism being the most common. This study provides evidence for the need of regular monitoring of thyroid function test among children with DS.
